# Clinical and epidemiologic characteristics of dengue and other etiologic agents among patients with acute febrile illness, Puerto Rico, 2012–2015

**DOI:** 10.1371/journal.pntd.0005859

**Published:** 2017-09-13

**Authors:** Kay M. Tomashek, Olga D. Lorenzi, Doris A. Andújar-Pérez, Brenda C. Torres-Velásquez, Elizabeth A. Hunsperger, Jorge Luis Munoz-Jordan, Janice Perez-Padilla, Aidsa Rivera, Gladys E. Gonzalez-Zeno, Tyler M. Sharp, Renee L. Galloway, Mindy Glass Elrod, Demetrius L. Mathis, M. Steven Oberste, W. Allan Nix, Elizabeth Henderson, Jennifer McQuiston, Joseph Singleton, Cecilia Kato, Carlos García Gubern, William Santiago-Rivera, Jesús Cruz-Correa, Robert Muns-Sosa, Juan D. Ortiz-Rivera, Gerson Jiménez, Ivonne E. Galarza, Kalanthe Horiuchi, Harold S. Margolis, Luisa I. Alvarado

**Affiliations:** 1 Dengue Branch, Division of Vector-Borne Diseases, Centers for Disease Control and Prevention (CDC), San Juan, Puerto Rico, United States of America; 2 Ponce Health Sciences University/Saint Luke's Episcopal Hospital, Ponce, Puerto Rico, United States of America; 3 Bacterial Special Pathogens Branch, Zoonoses and Select Agent Laboratory, CDC, Atlanta, Georgia, United States of America; 4 Polio and Picornavirus Laboratory Branch, Division of Viral Diseases, National Center for Immunization and Respiratory Diseases, CDC, Atlanta, Georgia, United States of America; 5 Rickettsial Zoonoses Branch, Division of Vector-Borne Diseases, CDC, Atlanta, Georgia, United States of America; 6 Saint Luke’s Episcopal Hospital, Guayama, Puerto Rico, United States of America; 7 Office of the Director, Division of Vector-Borne Diseases, CDC, Fort Collins, Colorado, United States of America; Jamia Millia Islamia, INDIA

## Abstract

Identifying etiologies of acute febrile illnesses (AFI) is challenging due to non-specific presentation and limited availability of diagnostics. Prospective AFI studies provide a methodology to describe the syndrome by age and etiology, findings that can be used to develop case definitions and multiplexed diagnostics to optimize management. We conducted a 3-year prospective AFI study in Puerto Rico. Patients with fever ≤7 days were offered enrollment, and clinical data and specimens were collected at enrollment and upon discharge or follow-up. Blood and oro-nasopharyngeal specimens were tested by RT-PCR and immunodiagnostic methods for infection with dengue viruses (DENV) 1–4, chikungunya virus (CHIKV), influenza A and B viruses (FLU A/B), 12 other respiratory viruses (ORV), enterovirus, *Leptospira* spp., and *Burkholderia pseudomallei*. Clinical presentation and laboratory findings of participants infected with DENV were compared to those infected with CHIKV, FLU A/B, and ORV. Clinical predictors of laboratory-positive dengue compared to all other AFI etiologies were determined by age and day post-illness onset (DPO) at presentation. Of 8,996 participants enrolled from May 7, 2012 through May 6, 2015, more than half (54.8%, 4,930) had a pathogen detected. Pathogens most frequently detected were CHIKV (1,635, 18.2%), FLU A/B (1,074, 11.9%), DENV 1–4 (970, 10.8%), and ORV (904, 10.3%). Participants with DENV infection presented later and a higher proportion were hospitalized than those with other diagnoses (46.7% versus 27.3% with ORV, 18.8% with FLU A/B, and 11.2% with CHIKV). Predictors of dengue in participants presenting <3 DPO included leukopenia, thrombocytopenia, headache, eye pain, nausea, and dizziness, while negative predictors were irritability and rhinorrhea. Predictors of dengue in participants presenting 3–5 DPO were leukopenia, thrombocytopenia, facial/neck erythema, nausea, eye pain, signs of poor circulation, and diarrhea; presence of rhinorrhea, cough, and red conjunctiva predicted non-dengue AFI. By enrolling febrile patients at clinical presentation, we identified unbiased predictors of laboratory-positive dengue as compared to other common causes of AFI. These findings can be used to assist in early identification of dengue patients, as well as direct anticipatory guidance and timely initiation of correct clinical management.

## Author summary

We conducted a prospective study of acute febrile illness (AFI) in Puerto Rico to better understand the etiology of AFI among all age groups in the tropics. Such findings could assist clinicians to identify disease-specific characteristics, which can then be used to initiate proper patient management. We enrolled 8,996 AFI patients and tested them for dengue viruses 1–4 (DENV 1–4) and 21 other pathogens. A pathogen was detected in 55% of patients, most frequently chikungunya virus (CHIKV, 18%), influenza A or B virus (FLU A/B, 12%), DENV 1–4 (11%), or another respiratory virus (ORV, 10%). Participants with dengue presented later after symptom onset and were hospitalized more often (47%) than patients with another etiology of AFI (27% with ORV, 19% with FLU A/B, and 11% with CHIKV). Predictors of patients with dengue differed by timing of presentation but included eye pain, nausea, and low white blood cell or platelet counts; negative predictors included symptoms of respiratory illness. By enrolling febrile patients at clinical presentation, we identified unbiased predictors of patients with dengue as compared to other common AFI. Findings can be used to diagnose dengue patients to provide early and appropriate clinical management.

## Introduction

As malaria incidence continues to decrease throughout the tropics, a new area of research has focused on identifying etiologies of non-malaria, acute febrile illness (AFI) [1, 2]. Knowledge is limited in this area, in large part because AFIs often have similar non-specific clinical presentations early in the clinical course when most patients are likely to present for care. In addition, rapid point-of-care diagnostics are often not readily available. Surveillance for AFIs, if done, is largely passive and relies on clinical identification of cases and voluntary case reporting. Therefore, burden of disease for the etiologic agents of AFI are likely underestimated. An improved understanding of the major causes of AFI is important to guide clinical management, develop diagnostics, inform public health policy, and direct prevention efforts [3].

In Mexico, South and Central America, and the Caribbean, AFI are common among patients of all age groups. In the last four decades, dengue, a mosquito-borne AFI caused by four genetically distinct dengue viruses (DENV 1–4), has become an increasingly common cause of AFI [4, 5]. The burden of dengue is thought to be less in Latin America than in Southeast Asia [6]; however, several studies have found that the incidence of dengue is likely underestimated in Latin America due to reliance on passive case surveillance [6–8]. Understanding region-specific etiologies of AFI and estimating the true incidence of dengue is necessary to plan large scale interventional trials for assessing the impact of mosquito control measures and vaccines. In addition, collecting clinical signs and symptoms from AFI patients of all ages with identified etiologic agents has utility in developing unbiased case definitions and identifying early clinical predictors to guide clinical management.

Prospective studies enrolling patients with AFI provide a methodology to systematically identify causes of AFI in a population and describe variation in the clinical course by patient age and etiologic agents. Since 2000, nine such studies have evaluated AFIs including dengue among both pediatric and adult patients [9–18]. While these studies are comparable in many ways, the studies differ in that several excluded either infants and young children [9–13, 16], older adults [12, 13], severe or hospitalized cases [13, 16], or cases with a known source of fever [9–12, 14]. In addition, most studies were conducted in low resource settings in Southeast Asia where malaria is still endemic [9, 10, 12–18]. In fact, two studies enrolled based on a potential participant meeting national eligibility criteria for malaria testing [12, 13], and two other studies excluded cases based on malaria blood smear positivity [14, 15].

In this manuscript, we describe a 3-year prospective study of AFI among all age groups that used a pre-defined diagnostic testing algorithm for DENV 1–4 and 21 other pathogens. We conducted this study in Puerto Rico, where malaria was eradicated in 1962 [19] and dengue has been endemic since the late 1960s [20]. We describe the frequency of dengue and other AFIs, and the distribution of these diseases in terms of person, place and seasonality. Last, we describe clinical predictors of dengue by timing of presentation compared to other AFIs.

## Materials and methods

### Study population

The study was conducted in southern Puerto Rico at Saint Luke’s Episcopal Hospital (SLEH)–Ponce, a 425-inpatient bed, tertiary care teaching hospital during May 7, 2012–May 6, 2015; and SLEH—Guayama, a 161-inpatient bed hospital during February 1, 2013–May 6, 2015. SLEH-Ponce is one of four hospitals serving 481,708 residents of Ponce and 11 neighboring municipalities [21]. SLEH-Ponce has about 50,000 emergency department (ED) visits and 11,000 inpatient admissions annually. SLEH-Guayama is one of two hospitals that serve 96,439 residents of Guayama and three adjacent municipalities. SLEH-Guayama has 35,000 ED visits and 6,000 inpatient admissions annually.

### Study enrollment and procedures

Patients presenting to the ED or as a direct hospital admission were eligible for enrollment if fever was present (defined by a body temperature of ≥38.0°C [oral] or ≥38.5°C [axillary]) or they reported a history of fever of ≤7-day duration. After informed consent was administered, vital signs, signs and symptoms of current AFI, history of exposures and chronic disease, and clinical laboratory results were recorded on an enrollment case report form (CRF). A physician examined the participants and recorded the clinical diagnosis on the CRF. Participants discharged to home after enrollment were asked to return 7–10 days post-illness onset (DPO). At the follow-up visit, a second completed CRF included a report of any healthcare services received and signs and symptoms experienced since enrollment. Participants admitted to the hospital upon enrollment had their hospital course summarized on a standardized data collection form that included treatment received, results of clinical laboratory and radiologic investigations, and disease manifestations.

### Ethics statement

Prior to enrollment, informed consent was administered in accordance with Puerto Rico law (Article 13, Section 13, Regulation 7617 of the Office of Patient Ombudsman, Act #194). Specifically, written informed consent was obtained from eligible adults >20 years old and emancipated minors 14–20 years old. Written informed assent was obtained from non-emancipated minors 14–20 years old and written informed consent was obtained from the parents or guardians. Verbal informed assent was obtained from children 7–13 years old and written informed consent was obtained from the parents or guardian, and the parents or guardian of children <7 years old. The Institutional Review Boards at the Centers for Disease Control and Prevention (CDC) and Ponce Health Sciences University approved the study protocol.

### Specimen collection

All study participants had blood (5 mL in EDTA, 7 mL whole blood), urine (15 mL), nasopharyngeal (NP), and oropharyngeal (OP) specimens collected at enrollment. Convalescent blood (5 mL in EDTA, 5 mL whole blood) and urine (10 mL) were collected at the follow-up visit or hospital discharge. NP and OP specimens were placed in a vial containing viral transport medium. Serum, blood, and urine specimens and inoculated vials were kept at 4°C until transported to CDC Dengue Branch (CDC-DB) in San Juan, Puerto Rico.

### Laboratory diagnostics

Molecular diagnostic testing for DENV 1–4, influenza A and B viruses (FLU A/B), and 12 other respiratory viruses (ORV) including adenovirus (AdV), human respiratory syncytial virus (HRSV), human metapneumovirus (HMPV), parainfluenza virus 1–4 (PIV-1–4), human rhinovirus (HRV), and four human coronaviruses (HCoV) (229E, OC43, NL63 and HKU1), was performed at CDC-DB. However, testing for HRV, PIV-2, PIV-4, and the four HCoV was discontinued after the first year because of low yield (i.e., only 1 PIV-2, 37 HCoV and 4 HCoV co-infections identified). In brief, RNA was extracted from NP and OP specimens and tested for ORV and FLU A/B viral genome by real time, reverse transcriptase-polymerase chain reaction assay (rRT-PCR) [22]. Serum specimens collected ≤6 DPO were tested by DENV-serotype specific rRT-PCR [23, 24], and those collected ≥4 DPO were tested by an antibody-capture enzyme-linked immunosorbent assay (MAC-ELISA) (InBios International, Inc., Seattle, WA)[25–27]. Beginning in May 2014, specimens collected ≤6 DPO were tested by CHIKV-specific real-time RT-PCR [28], and those collected ≥6 DPO were tested by anti-CHIKV MAC-ELISA [25]. Remaining serum, whole blood, and urine were stored at -70°C until shipped to CDC in Atlanta, Georgia.

At CDC, serum specimens collected ≤3 DPO were tested in the Picornavirus Laboratory by a pan-enterovirus real-time RT-PCR assay that targets the VP1 region [29]; positive specimens were sequenced. Paired serum specimens from enrollment and the follow-up visit or hospital discharge were tested for *Leptospira* spp., and *Burkholderia pseudomallei* at the Bacterial Special Pathogens Branch Laboratory. Specimens were tested by microscopic agglutination test (MAT) for 20 *Leptospira* reference antigens representing 17 serogroups [30]. All convalescent serum specimens were tested for presence of *Burkholderia pseudomallei* and *Leptospira* antibodies by an indirect hemagglutination assay (IHA) [31] and MAT respectively, and acute specimens were tested in cases for which the corresponding convalescent specimen was positive. The first 250 patients with *Leptospira spp*. and *Burkholderia pseudomallei* negative specimens and for which paired specimens were available were tested by IFA for *Rickettsia spp*., *Ehrlichia spp*., and *Coxiella spp*. at the Rickettsial Zoonoses Branch Laboratory. Whole blood and/or acute serum from cases with a reactive IFA were assessed for *C*. *burnetii*, *R*. *rickettsii*, *R*. *typhi*, *and/or E*. *chaffeensis* DNA by PCR.

### Etiologic definitions

A laboratory-positive dengue case had DENV nucleic acid or anti-DENV IgM detected in a single specimen. A laboratory-negative dengue case had no anti-DENV IgM detected in serum collected ≥6 DPO. A laboratory-positive influenza case was defined by presence of FLU A/B nucleic acid in a NP or OP specimen. Laboratory-positive HMPV, HRSV, ADENO, PIV-1, PIV-2, PIV-3, PIV-4, HRV, and HCoV cases had the respective viral nucleic acid present in a NP or OP specimen. A laboratory-positive leptospirosis case was defined by ≥4-fold increase in MAT titers in paired specimens, or MAT titer ≥800 in a single specimen. A laboratory-positive melioidosis case was defined by presence of *Burkholderia pseudomallei* nucleic acid in a clinical specimen and/or a ≥4-fold rise in IHA titer in paired specimens. A laboratory-positive enteroviral case was defined by presence of enterovirus nucleic acid in serum collected ≤3 DPO. A laboratory-positive ehrlichiosis case was defined by presence of *Ehrlichia chaffeensis* IgG reciprocal titer >1:128 by IFA, a ≥4-fold rise in IgG titer in paired serum specimens, or a positive PCR on an acute whole blood or serum specimen. A laboratory-positive Rickettsia case was defined by presence of *R*. *rickettsii* or *R*. *typhi* IgG titer >1:128 by IFA, a ≥4-fold rise in IgG titer in paired serum specimens, or a positive PCR in a whole blood or serum specimen. A laboratory-positive Coxiella case was defined by presence of *C*. *burnetii* IgG titer >1:128 by IFA, a ≥4-fold rise in IgG titer in paired serum specimens, or positive PCR on an acute whole blood or serum specimen.

### Clinical definitions

Leukopenia was defined as a white blood cell count ≤5,000 cells/μL. Thrombocytopenia was defined as a platelet count ≤100,000/μL. Severe hemoconcentration was defined by a hematocrit ≥20% above the U.S. population mean hematocrit for age and sex, and moderate hemoconcentration was defined by a hematocrit >97.5^th^ percentile for age and sex to less than the cut-off for severe hemoconcentration [32]. A skin bleed was defined by presence of skin bruising and/or petechiae. Mucosal bleeds included epistaxis, gingival bleed, hematemesis, melena, hematochezia, menorrhagia, or hematuria (>5 red blood cells per high powered field) in a male or non-menstruating female.

### Data analysis

Frequencies were calculated for demographic characteristics and medical history by study year. Clinical and laboratory features were compared by sex, age group, and laboratory diagnostic groups including infection with DENV, FLU A/B, ORV, and CHIKV. Differences in proportions were tested by applying the chi-square test, and medians were compared using the Mann-Whitney-Wilcox test. Bonferroni correction was used to account for simultaneous multiple comparisons. The Woolf test was performed to evaluate the homogeneity of odds ratio across DPO group for death among adult participants by sex, and the Mantel-Haenzel test was used to determine significance. Multiple imputation was used to predict an independent plausible value for missing values using generalized linear regression on non-missing variables to create 40 imputed complete data sets [33]. To identify predictors of laboratory-positive dengue as compared to all other AFI cases, stepwise Akaike Information Criterion (AIC) variable selection was used for each imputed data set. Variables retained at least once in the 40 models were included in a pooled logistic regression model [34]. Odds ratios (OR) and 95% confidence intervals (CI) were calculated for significant early (<3 DPO) and late (3–5 DPO) predictors. Data were analyzed using the “mi” and “MASS” packages from R software (V3.3.0, R Foundation for Statistical Computing, Vienna, Austria).

## Results

**Fig 1:**
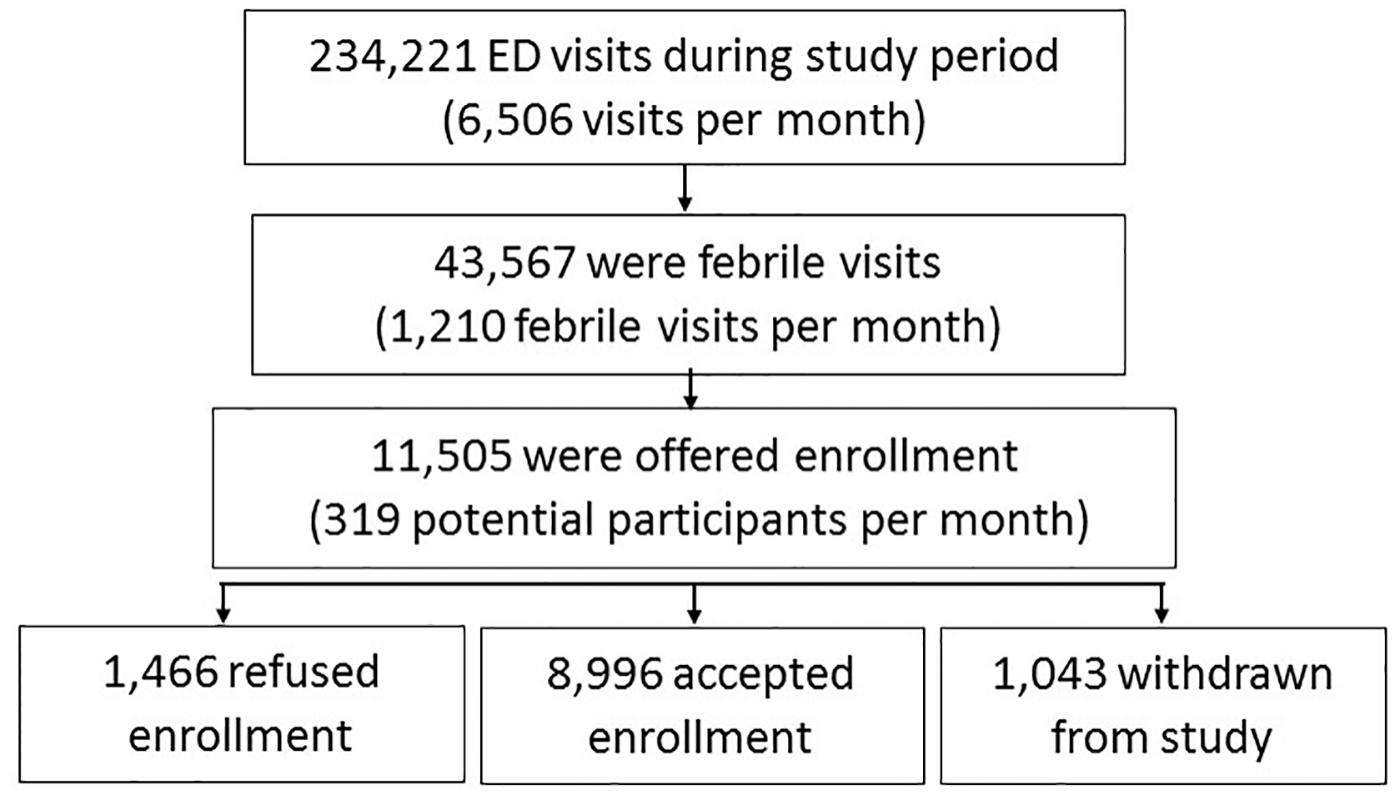
Study enrollment flow chart, acute febrile illness study, May 7, 2012–May 6, 2015, Puerto Rico.

During the study, sites recorded 234,221 ED visits of which 43,567 (18.6%) patients had fever or reported fever (Fig 1). Enrollment was offered to 11,505 AFI case-patients, and 10,039 (87.3%) gave their consent/assent to participate. However, 1,043 (10.4%) of those enrolled withdrew from the study or were withdrawn due to noncompliance with study enrollment procedures. Of the remaining 8,996 participants, 2,999 (33.3%) had follow-up forms completed and 24.9% had follow-up specimens collected. Half (50.3%) of the 8,996 participants were female, and the median age was 12.8 years (range 0–103) (Table 1). One-third (33.7%) of participants reported having a chronic medical condition; a higher proportion of participants enrolled in the first year reported a co-morbidity than those enrolled in subsequent years. The most common co-morbid conditions were asthma (18.6%), high blood pressure (10.7%), diabetes (7.5%), high cholesterol (4.7%), coronary heart disease (4.4%), and thyroid disease (4.2%). Participants resided in 49 of the 78 municipalities in Puerto Rico; however, most (76.3%) were residents of five municipalities: Ponce (43.0%), Guayama (15.6%), Juana Díaz (8.2%), Salinas (4.9%), and Villalba (4.6%).

**Table 1:** Characteristics of participants by year of enrollment, acute febrile illness study, May 7, 2012–May 6, 2015, Puerto Rico.

Participant Characteristics	Overall (n = 8,996)		First Year (n = 2,278)		Second Year (n = 3,110)		Third Year (n = 3,608)	
	Median	Range	Median	Range	Median	Range	Median	Range
**Age**	12.8	0.0–103.3	13.9 *	0.0–98.0	10.3 * ^‡^	0.0–103.3	14.1 ^‡^	0.0–97.3
	**N**	**%**	**n**	**%**	**n**	**%**	**n**	**%**
**Age group (years)**								
<1	735	8.2	152	6.7 *	291	9.4 *	292	8.1
1–4	1,984	22.1	424	18.6 * ^†^	781	25.1 * ^‡^	779	21.6 ^†^ ^‡^
5–9	1,292	14.4	339	14.9	465	15.0	488	13.5
10–19	1,666	18.5	566	24.8 * ^†^	513	16.5 *	587	16.3 ^†^
20–49	2,072	23.0	485	21.3	723	23.2	864	23.9
50+	1,247	13.9	312	13.7 * ^†^	337	10.8 * ^‡^	598	16.6 ^†^ ^‡^
**Female**	4,526	50.3	1,102	48.4	1,584	50.9	1,840	51.0
**Chronic medical conditions**								
One or more condition reported	3,036	33.7	851	37.4 * ^†^	1,026	33.0 *	1,159	32.1 ^†^
Asthma	1,673	18.6	489	21.5 ^†^	618	19.9 ^‡^	566	15.7 ^†^ ^‡^
Cancer	133	1.5	55	2.4 * ^†^	41	1.3 *	37	1.0 ^†^
Chronic obstructive pulmonary disease	71	0.8	35	1.5 * ^†^	23	0.7 *	13	0.4 ^†^
Coronary heart disease	393	4.4	136	6.0 * ^†^	117	3.8 *	140	3.9 ^†^
Diabetes	677	7.5	220	9.7 * ^†^	187	6.0 *	270	7.5 ^†^
High blood pressure	963	10.7	273	12.0 *	257	8.3 * ^‡^	433	12.0 ^‡^
High cholesterol	426	4.7	148	6.5 * ^†^	106	3.4 * ^‡^	172	4.8 ^†^ ^‡^
Immunodeficiency	75	0.8	23	1.0	35	1.1 ^‡^	17	0.5 ^‡^
Kidney disease	114	1.3	35	1.5	35	1.1	44	1.2
Liver disease	57	0.6	18	0.8	23	0.7	16	0.4
Sickle cell disease	62	0.7	26	1.1 ^†^	22	0.7	14	0.4 ^†^
Thyroid disease	377	4.2	118	5.2 *	115	3.7 *	144	4.0
**Municipality of residence**	**N**	**%**	**N**	**%**	**N**	**%**	**N**	**%**
Guayama	1,405	15.6	138	6.1 * ^†^	597	19.2 *	670	18.6 ^†^
Juana Diaz	742	8.2	212	9.3	252	8.1	278	7.7
Ponce	3,870	43.0	1,246	54.7 * ^†^	1,051	33.8 * ^‡^	1,573	43.6 ^†^ ^‡^
Salinas	437	4.9	56	2.5 * ^†^	181	5.8 *	200	5.5 ^†^
Villalba	410	4.6	100	4.4	176	5.7 ^‡^	134	3.7 ^‡^
Other	2,132	23.7	526	23.1 *	853	27.4 * ^‡^	753	20.9 ^‡^

* First year versus second year, significant difference in proportions (chi-square test) or medians (Mann-Whitney-Wilcoxon Test), with Bonferroni correction for multiple comparison (6). Significance level used: 0.05.

^†^ First year versus third year, significant difference in proportions or medians

^‡^ Second year versus third year, significant difference in proportions or medians

### Participant characteristics

Most (71.8%) participants were enrolled <3 DPO (median DPO at enrollment = 1, range: 0–8 days) (Table 2). The timing of presentation did not differ by sex but did differ by age, with a higher proportion of child participants (i.e., <20 years old) presenting <3 DPO than adult participants (74.9% child vs. 68.2% adult females, p <0.001; and 73.4% child vs. 67.4% adult males, p <0.001). One quarter (24.9%) of participants were admitted to the hospital at enrollment. Adult participants were less likely to be admitted than child participants; a higher proportion of female adult participants than male adult participants were sent home after enrollment (78.3% vs. 74.6% respectively, p <0.05). However, a higher proportion of male versus female adult participants died after enrollment (0.8% vs. 0.2% respectively), in fact, adult males were five times more likely to die than adult females when adjusting by DPO (OR = 5.4, CI: 1.5–19.0). There were no statistical significant differences between female and male participants <20 years old in terms of the timing of presentation and disposition. The most common signs and symptoms (aside from fever) at enrollment were tiredness/lethargy (73.5%), anorexia (65.0%), chills (64.5%), headache (64.3%), muscle, bone or back pain (60.0%), cough (53.4%), red conjunctiva (49.2%), rhinorrhea (49.1%), nausea (48.9%), and joint pain (48.9%).

**Table 2:** Clinical features of participants at enrollment, acute febrile illness study, May 7, 2012–May 6, 2015, Puerto Rico.

Clinical Features at Study Enrollment	All Participants		Female Participants (n = 4,526)				Male Participants (n = 4,470)			
			<20 years (n = 2,622)		20+ years (n = 1,904)		<20 years (n = 3,055)		20+ years (n = 1,415)	
	Median	Range	Median	Range	Median	Range	Median	Range	Median	Range
**Days post-illness onset (DPO)**	1	0.0–8.0	1.0 *	0.0–8.0	1.0 *	0.0–8.0	1.0 ^†^	0.0–8.0	1.0 ^†^	0.0–8.0
**DPO Group**	**N**	**%**	**N**	**%**	**N**	**%**	**N**	**%**	**N**	**%**
<3 days	6,458	71.8	1,964	74.9 *	1,299	68.2 *	2,241	73.4 ^†^	954	67.4 ^†^
3–5 days	2,193	24.4	586	22.3 *	498	26.2 *	708	23.2 ^†^	401	28.3 ^†^
6–8 days	345	3.8	72	2.7 *	107	5.6 *	106	3.5	60	4.2
**Disposition**										
Admitted	2,239	24.9	673	25.7 *	410	21.5 *	818	26.8 ^†^	338	23.9^†^
Died	15	0.2	0	0	3	0.2 ^‡^	0	0.0 ^†^	12	0.8 ^†^ ^‡^
Sent home	6,722	74.7	1,944	74.1 *	1,491	78.3* ^‡^	2,231	73.0	1,056	74.6 ^‡^
Transferred to other hospital	20	0.2	5	0.2	-	0.0 ^‡^	6	0.2 ^†^	9	0.6 ^†^ ^‡^

* Child versus adult participants within female group, significant difference in proportions (chi-square test) or (Mann-Whitney-Wilcoxon Test). Significance level used: 0.05. Mann-Whitney-Wilcoxon test was used to determine if distributions are identical.

^†^ Child versus adult participants within male group, significant difference in proportions or medians

^‡^ Female versus male participants within adult group, significant difference in proportions or medians

### Etiologic agents identified

**Fig 2:**
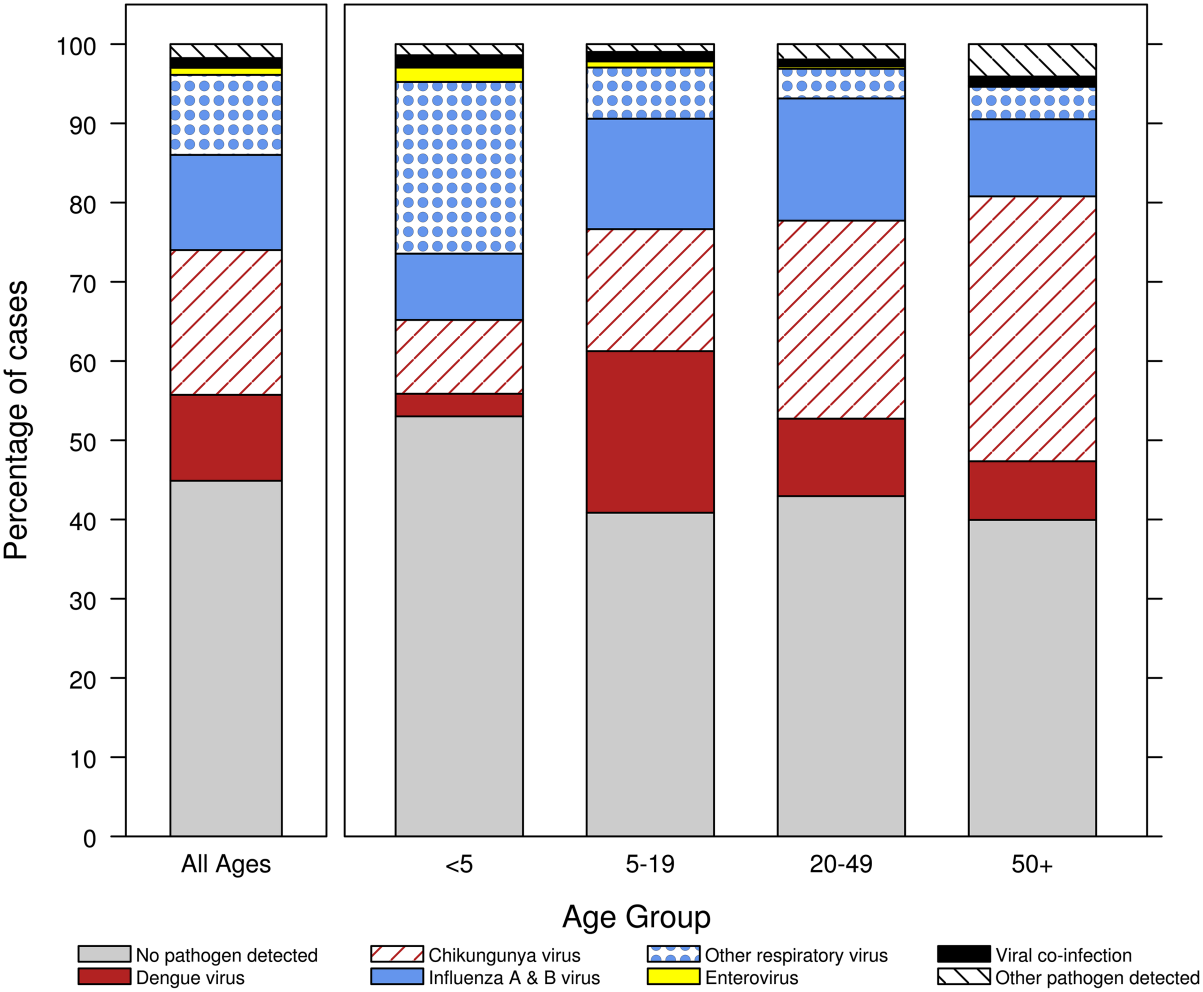
Number and proportion of enrolled participants by pathogens detected, overall and by age group, acute febrile illness study, May 7, 2012–May 6, 2015, Puerto Rico.

Slightly more than half (54.8%, 4,930) of the 8,996 participants had a pathogen detected (Fig 2). CHIKV was detected in 1,635 (18.2%) participants and was the most common pathogen detected, followed by FLU A/B (1,074, 11.9%), DENV 1–4 (970, 10.8%), and ORV (904, 10.3%). Most chikungunya (1,499, 91.7%) and dengue (685, 70.6%) cases were confirmed by RT-PCR. Among PCR-positive cases, DENV-1 was detected most frequently (645, 94.2%), followed by DENV-4 (38, 5.5%), and DENV-2 (2, 0.3%); no DENV-3 infections were identified. The majority (736, 68.5%) of influenza cases had FLU A virus detected. Among the ORV cases, adenovirus was detected most frequently (284, 31.4%), followed by RSV (175, 19.4%), HMPV (168, 18.6%), PIV-3 (138, 15.3%), PIV-1 (101, 11.2%), HCoV (37, 4.1%), and PIV-2 (1, 0.1%). Overall, enterovirus (80, 0.9%), leptospirosis (11, 0.1%), and melioidosis (2, 0.02%) cases were infrequently identified. Positive blood, urine or other culture, taken at the discretion of the site physician, were available for 145 (1.6%) participants. Co-infection was identified by molecular detection of two pathogens in 109 participants (Table 3). Co-infections most commonly occurred among participants infected with enterovirus (13/80, 16.3% of all enterovirus cases), followed by ORV (67/904, 7.4%), FLU A/B (46/1074, 4.3%), DENV (34/970, 3.5%), and CHIKV (27/1635, 1.7%).

**Table 3:** Co-infections by pathogens detected (n = 109), acute febrile illness study, May 7, 2012–May 6, 2015, Puerto Rico.

	Dengue Virus 1–4		Influenza A & B Virus		Other Respiratory Viruses*		Chikungunya Virus		Enterovirus	
	n	%	n	%	n	%	n	%	n	%
Dengue virus 1–4			7	15.2	23	28.8	0	0.0	3	23.1
Influenza A & B virus	7	20.6			23	28.8	8	29.6	6	46.2
Other respiratory viruses	23	67.6	23	50.0	13	16.3	17	63.0	4	30.8
Chikungunya virus	0	0.0	8	17.4	17	21.3			0	0.0
Enterovirus	3	8.8	6	13.0	4	5.0	0	0.0		
*Burkholderia pseudomallei*	1	2.9	2	4.3	0	0.0	0	0.0	0	0.0
*Leptospira* spp.	0	0.0	0	0.0	0	0.0	2	7.4	0	0.0
**Total**	**34**	**100.0**	**46**	**100.0**	**80**	**100.0**	**27**	**100.0**	**13**	**100.0**

* Other respiratory viruses include: human respiratory syncytial virus, human metapneumovirus, parainfluenza virus 1, parainfluenza virus 2, parainfluenza virus 3, parainfluenza virus 4, adenovirus, rhinovirus, and four human coronavirus.

The distribution of pathogens causing AFI varied by age (Fig 2). The proportion of chikungunya cases increased with age, accounting for 9.3% of all AFI cases in participants <5 years old versus 33.4% in participants ≥50 years old. In contrast, the contribution of ORV to AFI cases decreased with age, making up 21.6% of AFI cases in participants <5 years old, 6.4% in participants 5–19 years old, 3.7% in participants 20–49 years old, and 4.1% in participants ≥50 years old. Dengue was the most common cause of AFI in participants 5–19 years old, accounting for 20.3% of all cases; 2.8% of AFI cases in participants <5 years old were dengue, 9.8% in participants 20–49 years old, and 7.4% in participants ≥50 years old. The contribution of influenza was similar among age groups making up 8.4% of AFI cases in participants <5 years old, 13.9% in 5–19 years old, 15.4% in 20–49 years old, and 9.7% in ≥50 year-old participants.

### Epidemiologic characteristics

**Fig 3:**
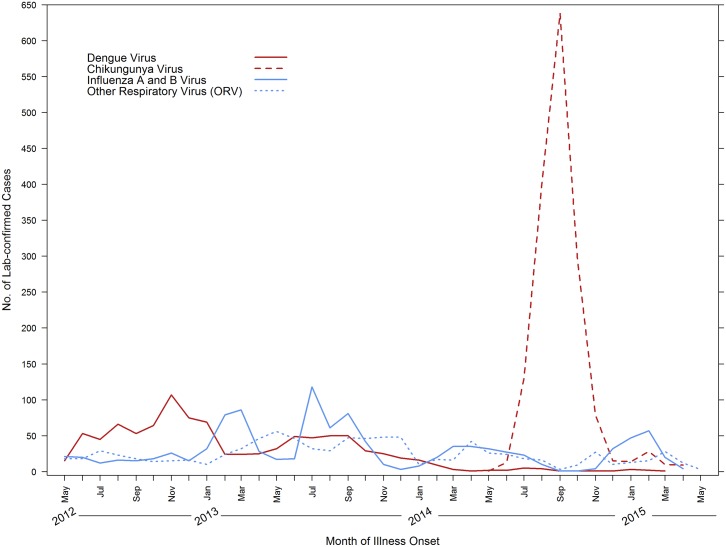
Number of laboratory-positive dengue, chikungunya, influenza and other respiratory viral illness by month of illness onset, acute febrile illness study, May 7, 2012–May 6, 2015, Puerto Rico.

Analysis of the temporal disease trends demonstrated that a dengue epidemic occurred in 2012 and continued through 2013, during which a total of 921 dengue cases were detected (Fig 3). In comparison, few (n = 49) dengue cases were detected in 2014 to the end of the study period in 2015. The first chikungunya case was detected in May of 2014, and was followed by a six-month outbreak during which 1,558 cases were detected. Few (n = 61) chikungunya cases were detected in 2015. A large bimodal influenza epidemic took place in 2013 with increased case detection in the dry months of January–April (n = 225), and during the rainy season, July–October (n = 302). Fewer influenza cases (n = 356) were detected in 2014 and 2015, and those detected occurred primarily in dry months with no obvious bimodal distribution. An increase in AFI cases due to ORV was detected at the same time influenza cases were detected, with the exception of 2013 when the peak time of ORV case detection appeared to follow that of influenza.

### Clinical features by etiologic agent

Subject demographics at enrollment differed by subsequent laboratory diagnosis (Table 4). A lower proportion of participants with dengue and ORV illness were females when compared with participants with chikungunya. Participants with ORV illness were significantly younger (median age = 3.2 years, p <0.001) than participants with dengue (15.4 years), chikungunya (24.3 years), or influenza (14.1 years). In contrast, the median age of participants with chikungunya was significantly greater than participants in all other diagnostic groups, and they were more likely to report having a chronic medical condition. A higher proportion of participants with dengue reported having a household member with dengue at enrollment than participants with other diagnoses (11.8% of dengue cases versus ≤5% in other diagnostic groups, p <0.001). Over half of all participants reported having mosquito bites in the 30 days before enrollment; however, a higher proportion of participants with chikungunya reported mosquito bites than participants with other laboratory diagnoses (73.9% versus <55% in other diagnostic groups, p<0.001).

**Table 4:** Characteristics and clinical features of participants at study enrollment by pathogen detected, acute febrile illness study, May 7, 2012–May 6, 2015, Puerto Rico.

Characteristics and Clinical Features at Study Enrollment	Dengue Virus 1–4 N = 970		Influenza A & B Virus N = 1,074		Other Respiratory Virus N = 904		Chikungunya Virus N = 1,635	
	N	%	N	%	N	%	N	%
**Female**	456	47.0 *	546	50.8	417	46.1 ^t^	865	52.9 * ^t^
**Has chronic medical condition**	290	29.9 *	356	33.1 ^ß^	264	29.2 ^t^	634	38.8 * ^ß^ ^t^
	**Median**	**Range**	**Median**	**Range**	**Median**	**Range**	**Median**	**Range**
**Age**	15.4 * ^‡^	0.0–88.9	14.1 ^ß^ ^£^	0.1–92.0	3.2 ^‡^ ^t^ ^£^	0.1–90.3	24.3 * ^ß^ ^t^	0.0–97.3
**Days post-illness onset (DPO)**	3.0 * ^†^ ^‡^	0.0–8.0	1.0 ^†^ ^ß^	0.0–8.0	2.0 ^‡^ ^t^	0.0–8.0	1.0 * ^ß^ ^t^	0.0–7.0
**DPO group**	**N**	**%**	**N**	**%**	**N**	**%**	**N**	**%**
<3 days	348	35.9 * ^†^ ^‡^	778	72.4 ^†^ ^ß^	630	69.7 ^‡^ ^t^	1,390	85.0 * ^ß^ ^t^
3–5 days	543	56.0 * ^†^ ^‡^	268	25.0 ^†^ ^ß^	246	27.2 ^‡^ ^t^	209	12.8 * ^ß^ ^t^
6–8 days	79	8.1 * ^†^ ^‡^	28	2.6 ^†^	28	3.1 ^‡^	36	2.2 *
**Exposure history**								
House member diagnosed with dengue	114	11.8 * ^†^ ^‡^	54	5.0 ^†^	28	3.1 ^‡^	81	5.0 *
Mosquito bites in last month	505	52.1 *	549	51.1 ^ß^	486	53.8 ^t^	1,208	73.9 * ^ß^ ^t^
**Disposition**								
Admitted	452	46.6 * ^†^ ^‡^	202	18.8 ^†^ ^ß^ ^£^	247	27.3 ^‡^ ^t^ ^£^	183	11.2 * ^ß^ ^t^
Died	1	0.1	2	0.2	1	0.1	2	0.1
Sent home	514	53.0 * ^†^ ^‡^	868	80.8 ^†^ ^ß^ ^£^	654	72.3 ^‡^ ^t^ ^£^	1,449	88.6 * ^ß^ ^t^
Transferred to other hospital	3	0.3	2	0.2	2	0.2	1	0.1
**Signs and symptoms**								
Chills	753	77.6 * ^†^ ^‡^	772	71.9 ^†^ ^£^	457	50.6 ^‡^ ^t^ ^£^	1,163	71.1 * ^t^
Sign of poor circulation	482	49.7 * ^†^ ^‡^	449	41.8 ^†^ ^ß^ ^£^	303	33.5 ^‡^ ^£^	519	31.7 * ^ß^
Skin rash	443	45.7 * ^†^ ^‡^	124	11.5 ^†^ ^ß^ ^£^	148	16.4 ^‡^ ^t^ ^£^	991	60.6 * ^ß^ ^t^
Facial and/or neck erythema	518	53.4 ^†^ ^‡^	360	33.5 ^†^ ^ß^	277	30.6 ^‡^ ^t^	933	57.1 ^ß^ ^t^
Pruritic skin	244	25.2 * ^†^ ^‡^	90	8.4 ^†^ ^ß^	86	9.5 ^‡^ ^t^	511	31.3 * ^ß^ ^t^
Red conjunctiva	498	51.3 *	613	57.1 ^£^	419	46.3 ^t^ ^£^	945	57.8 * ^t^
Eye pain	537	55.4 * ^†^ ^‡^	506	47.1 ^†^ ^£^	219	24.2 ^‡^ ^t^ ^£^	750	45.9 * ^t^
Headache	793	81.8 * ^†^ ^‡^	780	72.6 ^†^ ^£^	401	44.4 ^‡^ ^t^ ^£^	1,167	71.4 * ^t^
Muscle/bone/back pain	724	74.6 * ^†^ ^‡^	698	65.0 ^†^ ^ß^ ^£^	287	31.7 ^‡^ ^t^ ^£^	1,384	84.6 * ^ß^ ^t^
Joint pain	561	57.8 * ^†^ ^‡^	553	51.5 ^†^ ^ß^ ^£^	207	22.9 ^‡^ ^t^ ^£^	1,324	81.0 * ^ß^ ^t^
Red/swollen joints	116	12.0 * ^‡^	99	9.2 ^ß^ ^£^	37	4.1 ^‡^ ^t^ ^£^	710	43.4 * ^ß^ ^t^
Any bleeding	378	39.0 * ^†^ ^‡^	204	19.0 ^†^ ^ß^	158	17.5 ^‡^ ^t^	793	48.5 * ^ß^ ^t^
Skin bleeding	250	25.8 * ^†^ ^‡^	69	6.4 ^†^ ^ß^	72	8.0 ^‡^ ^t^	645	39.4 * ^ß^ ^t^
Mucosal bleeding	193	19.9 ^†^ ^‡^	150	14.0 ^†^	99	11.0 ^‡^ ^t^	287	17.6 ^t^
Tiredness, lethargy	828	85.4 ^†^ ^‡^	835	77.7 ^†^ ^£^	551	61.0 ^‡^ ^t^ ^£^	1,329	81.3 ^t^
Nervousness, anxiety	323	33.3 ^‡^	343	31.9 ^£^	178	19.7 ^‡^ ^t^ ^£^	541	33.1 ^t^
Irritability	267	27.5	270	25.1 ^ß^	269	29.8	503	30.8 ^ß^
Dizziness	579	59.7 * ^†^ ^‡^	437	40.7 ^†^ ^£^	157	17.4 ^‡^ ^t^ ^£^	709	43.4 * ^t^
Cough	360	37.1 * ^†^ ^‡^	941	87.6 ^†^ ^ß^ ^£^	701	77.5 ^‡^ ^t^ ^£^	430	26.3 * ^ß^ ^t^
Rhinorrhea	258	26.6 ^†^ ^‡^	843	78.5 ^†^ ^ß^ ^£^	650	71.9 ^‡^ ^t^ ^£^	445	27.2 ^ß^ ^t^
Sore throat	324	33.4 * ^†^ ^‡^	613	57.1 ^†^ ^ß^ ^£^	430	47.6 ^‡^ ^t^ ^£^	368	22.5 * ^ß^ ^t^
Anorexia	746	76.9 * ^†^ ^‡^	755	70.3 ^†^ ^ß^	593	65.6 ^‡^ ^t^	935	57.2 * ^ß^ ^t^
Nausea	637	65.7 * ^†^ ^‡^	545	50.7 ^†^ ^ß^ ^£^	396	43.8 ^‡^ ^£^	709	43.4 * ^ß^
Abdominal pain	541	55.8 * ^†^ ^‡^	471	43.9 ^†^ ^ß^ ^£^	298	33.0 ^‡^ ^£^	516	31.6 * ^ß^
Vomiting (3 or more episodes in day)	247	25.5 *	237	22.1 ^ß^	211	23.3 ^t^	226	13.8 * ^ß^ ^t^
Diarrhea	357	36.8 * ^†^ ^‡^	242	22.5 ^†^ ^ß^	239	26.4 ^‡^ ^t^	295	18.0 * ^ß^ ^t^
**Clinical Laboratory**								
Moderate hemoconcentration	37	3.9 *	25	2.5 ^ß^	22	2.6 ^t^	14	0.9 * ^ß^ ^t^
Severe hemoconcentration	10	1.1 *	2	0.2	4	0.5	3	0.2 *
Thrombocytopenic	355	36.6 * ^†^ ^‡^	23	2.1 ^†^	13	1.4 ^‡^	34	2.1 *
Leukopenic	716	73.8 * ^†^ ^‡^	293	27.3 ^†^ ^ß^ ^£^	84	9.3 ^‡^ ^t^ ^£^	344	21.0 * ^ß^ ^t^
	**Median**	**Range**	**Median**	**Range**	**Median**	**Range**	**Median**	**Range**
Platelet count (x 10^3)	121.0 * ^†^ ^‡^	10.0–511.0	210.0 ^†^ ^£^	15.6–610.0	265.0 ^‡^ ^t^ ^£^	28.9–597.0	215.0 * ^t^	23.0–635.0
White blood cells (x 10^3)	3.4 * ^†^ ^‡^	1.0–24.0	6.5 ^†^ ^£^	1.1–24.6	9.3 ^‡^ ^t^ ^£^	1.0–32.1	6.7 * ^t^	1.7–29.6

* Dengue virus versus Chikungunya virus, significant difference in proportions (Chi-square test) or median (Mann-Whitney-Wilcoxon test). Significance level used: 0.05 with Bonferroni correction for multiple comparisons (6)

^†^ Dengue virus versus Influenza A and B virus, significant difference in proportions or median.

^‡^ Dengue Virus versus other respiratory virus, significant difference in proportions or median.

^ß^ Chikungunya virus versus Influenza A and B virus, significant difference in proportions or median.

^t^ Chikungunya virus versus other respiratory virus, significant difference in proportions or median.

^£^ Influenza A and B virus versus other respiratory virus, significant difference in proportions or median.

Clinical presentation and disposition varied by laboratory diagnostic group (Table 4). Participants with laboratory-positive dengue presented later (median = 3 days), and a higher proportion were admitted at enrollment than participants with other laboratory diagnoses; nearly half (46.6%) of dengue cases were admitted compared with 27.3% of participants with ORV illness, 18.8% with influenza, and 11.2% with chikungunya. A significantly higher proportion of participants with dengue had chills, signs of poor circulation, eye pain, headache, dizziness, anorexia, nausea, abdominal pain, and diarrhea at enrollment than participants with influenza, ORV illness or chikungunya. A higher proportion of participants with dengue versus these other diagnoses had thrombocytopenia and leukopenia. Compared to influenza and ORV illness cases, a significantly higher proportion of dengue cases had a skin rash, pruritic skin, any bleeding, a skin bleed, and muscle, bone, back, and joint pain, whereas a higher proportion of chikungunya versus dengue cases had these findings. A higher proportion of dengue versus influenza and ORV illness cases had facial and/or neck erythema and mucosal bleeding. In contrast, a significantly higher proportion of participants with influenza and ORV illness than dengue had cough, rhinorrhea, and sore throat.

### Predictors by timing of presentation and age

Among 6,349 participants who presented early (<3 DPO) in the clinical course, leukopenia, thrombocytopenia, headache, eye pain, nausea, and dizziness were significant positive predictors of laboratory-positive dengue as compared to all other AFI cases across all age groups (Table 5). Presence of rhinorrhea and irritability predicted non-dengue AFI. Age group had a statistically significant effect on multiple predictors (Table 6). Rash was a positive early predictor of dengue among participants <5 years old, and being male was a positive predictor among adults 20–49 years old. Chills and cough were positive predictors for those >50 years old while cough was a negative predictor among those <20 years old. Muscle, bone or back pain was a negative predictor in those >50 years old. Pruritic skin as a predictor varied by age group, but most significantly between the <5 and 50+ year-old groups.

**Table 5:** Early predictors of laboratory-positive dengue versus all other acute febrile illnesses for all ages, acute febrile illness study, May 7, 2012–May 6, 2015, Puerto Rico*.

Positive Predictor	Adjusted Odds Ratio (aOR)	95% Confidence Interval (CI)
Leukopenia	6.61	5.06–8.63
Thrombocytopenia	3.66	2.26–5.93
Headache	1.55	1.07–2.24
Eye pain	1.47	1.14–1.93
Nausea	1.45	1.12–1.89
Dizziness	1.44	1.09–1.91
**Negative Predictor**		
Rhinorrhea	0.61	0.46–0.81
Irritability	0.70	0.52–0.93

* Table includes data from 6,349 participants of all ages presenting <3 days post-illness onset including 348 dengue cases.

**Table 6:** Early predictors of laboratory-positive dengue versus all other acute febrile illnesses by age group, acute febrile illness study, May 7, 2012–May 6, 2015, Puerto Rico.

Age Group								
Predictor	<5 years old (43 dengue of 2,106)		5–19 years old (208 dengue of 2,022)		20–49 years old (63 dengue of 1,396)		50+ years old (34 dengue of 825)	
	aOR	95% CI	aOR	95% CI	aOR	95% CI	aOR	95% CI
Chills	0.72	0.39–1.36	1.41	0.97–2.07	1.59	0.73–3.43	3.14*	**1.02–9.60**
Cough	**0.34**†	**0.17–0.67**	**0.52**†	**0.36–0.74**	0.58	0.32–1.04	2.78*	**1.27–6.09**
Muscle/bone/back pain	0.69	0.34–1.42	0.77	0.53–1.11	0.52	0.22–1.27	**0.18**†	**0.07–0.47**
Rash	2.10*	**1.05–4.18**	1.12	0.78–1.63	0.62	0.33–1.19	0.39	0.12–1.27
Male	0.65	0.36–1.19	1.27	0.92–1.75	2.37*	**1.37–4.10**	1.13	0.55–2.34
Pruritic skin ¥	1.74	0.71–4.28	0.74	0.45–1.21	1.48	0.76–2.90	0.27	0.07–1.06

* Significant positive predictor

^†^ Significant negative predictor

^¥^ Age significantly affects predictor across groups.

Among the 2,146 participants who presented 3–5 DPO, thrombocytopenia, leukopenia, facial and/or neck erythema, nausea, eye pain, signs of poor circulation, and diarrhea were significant positive predictors of dengue across all age groups (Table 7). Presence of rhinorrhea, red conjunctiva and cough predicted non-dengue AFI. Again, age group significantly affected multiple predictors (Table 8). Abdominal pain was a positive predictor for participants 20–49 years old. Red and/or swollen joints was a positive predictor among participants <5 years old but a predictor of non-dengue AFI among participants ≥50 years old. Leukopenia was a significant positive predictor across all age groups, but to varying degrees. Chills; muscle, bone, back and joint pain; and any bleeding as predictors varied depending on the age group.

**Table 7:** Late predictors of laboratory-positive dengue versus all other acute febrile illnesses for all ages, acute febrile illness study, May 7, 2012–May 6, 2015, Puerto Rico*.

Positive Predictor	Adjusted Odds Ratio (aOR)	95% Confidence Interval (CI)
Thrombocytopenia	5.36	3.73–7.71
Facial/neck erythema	1.74	1.26–2.38
Nausea	1.71	1.20–2.44
Eye pain	1.66	1.19–2.30
Poor circulation†	1.43	1.04–1.96
Diarrhea	1.40	1.02–1.92
**Negative Predictor**		
Rhinorrhea	0.30	0.21–0.42
Red conjunctiva	0.68	0.49–0.95
Cough	0.68	0.49–0.94

* Table includes data from 2,146 participants of all ages presenting 3–5 days post-illness onset including 543 dengue cases.

^†^ Sign of poor circulation included report of pale cold skin, and/or blue lips and/or skin.

**Table 8:** Late predictors of laboratory-positive dengue versus all other acute febrile illnesses by age group, acute febrile illness study, May 7, 2012–May 6, 2015, Puerto Rico.

Age Group								
Predictor	<5 years old (28 dengue of 482)		5–19 years old (350 dengue of 783)		20–49 years old (117 dengue of 544)		50+ years old (48 dengue of 337)	
	aOR	95% CI	aOR	95% CI	aOR	95% CI	aOR	95% CI
Leukopenia ¥	5.01*	**2.21–11.35**	15.30*	**9.24–25.33**	15.35*	**7.90–29.80**	7.57*	**3.51–16.36**
Red/swollen joints	3.45*	**1.16–10.29**	0.87	0.46–1.68	0.39†	0.18–0.83	**0.22**†	**0.08–0.64**
Abdominal pain	0.46	0.18–1.17	1.21	0.76–1.92	2.56*	**1.39–4.72**	0.83	0.37–1.84
Chills ¥	0.60	0.25–1.44	1.68	1.00–2.83	1.33	0.67–3.12	1.32	0.44–3.98
Muscle/bone/back pain ¥	2.41	0.92–6.29	0.98	0.60–1.60	0.90	0.31–2.61	0.54	0.16–1.80
Any bleeding ¥	1.85	0.82–4.18	1.40	0.90–2.17	0.66	0.37–1.18	0.42	0.18–1.01

* Significant positive predictor

^†^ Significant negative predictor

^¥^ Age significantly affects predictor across groups.

## Discussion

As a clinical syndrome, AFIs are a diagnostic challenge for clinicians especially early in the clinical course when anticipatory guidance and supportive care may pre-empt medical complications. Our study identified the AFI etiology in over half (55%) of participants and most were infected with one of nine viral pathogens. This detection frequency was higher than that of other recent prospective AFI studies that tested for multiple pathogens (55% versus 36–41%) [9–12, 15]. This difference may in part be explained by the greater contribution of chikungunya in our study than in the other studies that tested for this pathogen [9, 10, 17]. However, unlike other studies, we were unable to detect any evidence of disease caused by *Rickettsia* or *Coxiella* spp., which made up 4–13% of all AFIs in other studies [10–13]; and unlike other areas, malaria [9, 11–13, 18] and typhoid fever [9, 10, 12, 13, 15] were not part of our diagnostic algorithm as they are only occasionally detected among travelers returning to Puerto Rico.

While DENV was not as commonly identified as CHIKV or FLU A/B, participants with dengue were more likely to be admitted to the hospital at enrollment. The proportion of AFI cases with dengue in our study was comparable to other recent studies which found 4–9% of AFI cases had dengue [9–13, 15–18]. One exception to this was a study that found 34% of AFI patients had dengue; however, the study’s eligibility criteria likely enhanced enrollment of dengue cases [14]. In our study, dengue incidence varied by age group with a nearly a 10-fold difference between participants <5 years old and 10–19 years old (3% versus 27%), which may be due to differences in likelihood of seeking medical care in primary versus secondary DENV infections [35]. Of note, 6% of participants ≥65 years old had dengue as a cause of AFI, a finding comparable to a Puerto Rico study in which 5% of 17,666 laboratory-positive dengue cases detected by surveillance were ≥65 years old [36]. In contrast, other recent prospective studies [37, 38] and a cross-sectional serosurvey [39] conducted in other dengue endemic countries found few, if any, symptomatic dengue cases among older participants. Whether this is due to a lower force of infection in Puerto Rico, immunosenescence, evolution of genotypes/strains of DENV, differences in prevalence of underlying chronic disease or health care seeking behavior in Puerto Rico, or lack of life-long homotypic immunity is not known [40–42]. However, DENV-1 has been in circulation in Puerto Rico since the 1970s and involved in every major outbreak since [35, 43].

Chikungunya, the most commonly identified AFI overall, was least likely to result in hospital admission, although two male participants with CHIKV infection died. These cases were older individuals (>75 years old) who had underlying co-morbidities which may have complicated their clinical course. Nonetheless, since autopsy was not performed for either case, ascertaining whether CHIKV infection played a role in either fatality is difficult. However, in our study chikungunya was disproportionally identified among older participants, with positivity increasing from <10% of pre-school aged children to about one-third of participants ≥50 years old. This pattern of disease has been seen in other areas with recent CHIKV emergence [18], and may be due to older individuals having an increased likelihood of complications due to preexisting co-morbidities [44, 45].

Co-infections confirmed by molecular assays were detected among 1% of our participants, most commonly involving enteroviral or ORV infections; less than one-third of all co-infections included a DENV or CHIKV infection. Another recent prospective study found that 1% of AFI participants had co-infections involving molecularly diagnosed dengue or influenza, malaria, and positive blood culture [9]. Interestingly, we did not detect any co-infections involving CHIKV and DENV. An analysis of island-wide surveillance data from Puerto Rico during the same time period found only one CHIKV/DENV co-infection among approximately 1,000 specimens tested by RT-PCR for both DENV and CHIKV [46]. These findings are consistent with another prospective AFI study conducted in Sri Lanka [17]. Although a recent study has shown that *Aedes aegypti* can be infected with as many as three arboviruses simultaneously and can likely transmit these viruses to humans [47], the frequency of co- or tri-infection of mosquitoes in the wild depends upon the geographic spread and degree of circulation of each virus. During our study, DENV transmission decreased significantly before CHIKV transmission peaked, making co-infections less likely. In addition, in Puerto Rico, where *Aedes aegypti* is the sole vector for CHIKV and DENV, viral interaction and viral interference within the mosquito may reduce the likelihood of co-infection [48–50]. However, RT-PCR positive DENV/CHIKV co-infections have been documented at higher rates in five countries [51].

We identified differences in clinical predictors of laboratory-positive dengue by timing of presentation and age group highlighting the importance of considering these factors when developing prediction algorithms for clinical management [52–60]. We found, as have others [61], that even early (<3 DPO) in the clinical course leukopenia and thrombocytopenia are predictive of dengue across all age groups, and thrombocytopenia strengthened as a predictor over time. In our study, headache and eye pain were the only “aches and pains” that were predictive of dengue for all age groups [62]. Eye pain was a predictor early and later in the clinical course, a finding consistent with pediatric [63] and adult [56] prospective cohort studies, as well as a surveillance study conducted in Puerto Rico [52]. We also found that rash among children <5 years old presenting early and erythema on the face and/or neck in all age groups presenting 3–5 DPO, were positive predictors of dengue. While the presence of skin rash has been found to be a predictor of dengue in several prospective studies [61], few studies have evaluated erythema as a predictor [18, 64, 65]. Last, like other prospective studies [56, 66], we found that nausea is an early predictor for dengue. We were also able to show that adults aged 20–49 years presenting 3–5 DPO were more likely to have abdominal pain than those with other AFIs, and dengue cases of all ages presenting 3–5 DPO were also more likely to have diarrhea and poor circulation in addition to nausea, findings that lend support to the idea that warning signs for severe dengue develop after the early phase of the illness.

Our study, which enrolled all patients presenting with fever regardless of age, sex, or clinical characteristics, may be limited in generalizability. The study was conducted in southern Puerto Rico which may differ from neighboring islands and other parts of the island with regard to population demographics, preexisting immunity to DENV and other flaviviruses, and exposure to infections. Second, while we enrolled nearly 600 older adults (≥65 years old), we were unable to adequately evaluate predictors of dengue among this population because we had only 36 dengue cases and most presented early in the clinical course. Last, we did not systematically collect stool and test for potential enteric pathogens, and bacterial infections were likely under recognized because blood cultures were only done on patients in whom sepsis was suspected.

While our study identified an etiology in over half of all AFI cases, the etiology of 45% of AFI remained unknown even after extensive testing and the majority of diagnosed cases were caused by one of nine viral pathogens that typically do not require empiric therapy. In fact, we were unable to find any cases of *Rickettsia spp*., *Ehrlichia spp*., and *Coxiella spp*., and only sporadic cases of melioidosis and leptospirosis were identified. Our findings demonstrate that dengue is not only one of the leading causes of AFI in Puerto Rico, but results in greater morbidity than other AFIs as measured by need for hospitalization. Moreover, dengue affects people of all ages including older adults who may be at higher risk of developing medical complications. Clinicians should include dengue on the differential diagnosis of AFI among older adults so that timely anticipatory guidance can be offered. We found that the presence of leukopenia and thrombocytopenia were the best predictors of dengue in both time periods overall and for all age groups. Our findings suggest that eye pain should be reevaluated as a predictor of dengue. Future studies should focus on improving clinical diagnosis of AFI including dengue by timing of presentation and age of the patient.

## Supporting information

S1 Checklist STROBE statement. (DOCX)
